# Inactivated influenza vaccine effectiveness among department of defense beneficiaries aged 6 months-17 years, 2016–2017 through 2019–2020 influenza seasons

**DOI:** 10.1371/journal.pone.0256165

**Published:** 2021-08-27

**Authors:** Wenping Hu, Laurie S. DeMarcus, Paul A. Sjoberg, Anthony S. Robbins

**Affiliations:** 1 The Department of Defense Global Emerging Infections Surveillance Branch, Armed Forces Health Surveillance Division, WPAFB, OH, United States of America; 2 JYG Innovations, LLC, Dayton, OH, United States of America; National Center for Global Health and Medicine, JAPAN

## Abstract

A test-negative case-control study was conducted to assess inactivated influenza vaccine effectiveness (VE) in children aged 6 months-17 years. The database was developed from the US Department of Defense Global Respiratory Pathogen Surveillance Program over four consecutive influenza seasons from 2016 to 2020. A total of 9,385 children including 4,063 medically attended, laboratory-confirmed influenza-positive cases were identified for VE analysis. A generalized linear mixed model with logit link and binomial distribution was used to estimate the VE. The adjusted VE for children was 42% [95% confidence interval (CI): 37–47%] overall, including 55% (95% CI: 47–61%) for influenza A(H1N1)pdm09, 37% (95% CI: 28–45%) for influenza A(H3N2), and 49% (95% CI: 41–55%) for influenza B. The analysis by age groups indicated that the adjusted VE in children aged 6 months-4 years was higher against influenza A(H1N1)pdm09 and influenza B, and comparable against influenza A(H3N2), compared to those in children aged 5–17 years. Further age-stratified analysis showed that the VE against any types of influenza was low and non-significant for children aged 6–11 months (33%; 95% CI:-2-56%), but it was high (54%; 95% CI: 34–67%) in children aged 12–23 months, and then declined linearly with increasing age. In conclusion, the inactivated influenza vaccination was moderately effective against influenza infection, based on the analysis from a large number of children aged 6 months-17 years over multiple influenza seasons.

## Introduction

Seasonal influenza primarily causes human respiratory disease in all ages. Children, particularly those younger than 5 years of age, are at high risk of influenza infection and severe influenza-related complications including hospitalization and death [1, 2]. The disease burden due to influenza infection is substantial. It was estimated that over six influenza seasons (2010–2011 through 2015–2016) in the United States, the total numbers of influenza-related symptomatic illnesses and hospitalizations among children have ranged from 2.8 to 10.7 million, and 11,000 to 45,000, respectively [3]. Moreover, influenza imposes a considerable economic burden to the healthcare system and society. Putri et al. [4] estimated the annual economic burden of influenza among children in the United States was $2.3 billion, including direct medical cost of $0.7 billion (30.8%) and indirect costs of $1.6 billion (69.2%).

Influenza vaccination is considered the most effective way to protect against influenza viruses, combat influenza infection and lessen disease severity. The American Academy of Pediatrics recommends routine influenza immunization of all children without medical contraindications, starting at 6 months of age [5]. Every year, the Department of Defense (DoD) Global Respiratory Pathogen Surveillance (DoDGRS) Program performs routine respiratory pathogen surveillance among DoD service members and their beneficiaries, allowing annual estimates of influenza vaccine effectiveness (VE). Annual estimates of VE are necessary as the circulating influenza viruses differ from year to year. Nevertheless, in these previous DoDGRS studies [6–9], the VE has been assessed using data acquired in a single influenza season, in which all patients were categorized as children (6 months-17 years of age), adults (18–64 years of age), and the elderly(≥65 years of age). Sample sizes might be too small to be able to accurately estimate VE within age categories, such as children aged <2 years. However, combining available data over multiple influenza seasons allows to provide pooled VE estimate by influenza type/subtype in age-specific category. The objective of our study was to use DoDGRS data to evaluate the effectiveness of inactivated influenza vaccine (IIV) against medically attended, laboratory-confirmed influenza in children 6 months to 17 years of age among DoD beneficiaries in the United States seeking outpatient care over four influenza seasons (2016–2017 through 2019–2020).

## Methods

### Study population

We used DoDGRS data over four influenza seasons from 2016–2017 to 2019–2020. In DoDGRS, all patients seeking outpatient medical care for influenza-like illness (ILI) clinical condition were selected. ILI was defined if the patient has (1) a fever greater than or equal to 38°C and a cough or sore throat which presents within 72 hours after illness onset, or (2) physician determined ILI. Respiratory specimens were collected by nasopharyngeal wash or nasopharyngeal swab from ILI patients, and subject to testing via a multiplex respiratory pathogen panel, reverse transcription polymerase chain reaction (RT-PCR) and/or viral culture. Thus, the influenza viruses and other respiratory pathogens were identified and confirmed. Influenza virus type and subtype/lineage were identified when influenza virus tested positive. Patients who had received at least one influenza vaccine dose 14 days or more before the onset of an ILI, were considered vaccinated. Patients who otherwise had not received vaccination before the onset of an ILI, were considered unvaccinated. Vaccination status was verified through the records from the DoD Electronic Immunization Tracking System or self-reported questionnaire for each patient. Patients with an unknown vaccination status or type, or vaccinated <14 days prior to the onset of an ILI were excluded. Vaccine type in the present study was limited to IIV; thus, patients who received influenza vaccines other than IIV were excluded.

The patients in the present study were limited only to those aged 6 months-17 years from the DoDGRS program sentinel or participating sites in the United States.

### Age stratification

Age was classified as two main groups: 6 months-4 years age group, including two divided age subgroups (6–23 months and 2–4 years); and 5–17 years age group including three divided age subgroups (5–8 years, 9–12 years, and 13–17 years). The rationale for age classification was based on the fact that children younger than 5 years of age are at higher risk for influenza infection and severe influenza-related complications [1, 2]. In addition, it was of interest to evaluate VE against influenza viruses in children under 2 years old with extra age stratification. Thus, children aged <2 years were further stratified into two age strata (6–11 months and 12–23 months).

### Statistical analysis

Prior to data assembly over four influenza seasons, a range of surveillance weeks in each influenza season was limited to November to April of the following year for the VE analysis, when approximately 10% or greater influenza positivity rate occurred, with an aim to minimize any potential bias due to high ratio of influenza negative to influenza positive that would typically occur earlier or later in the influenza season.

All data was combined by influenza season and performed analysis using generalized linear mixed model (GLMM) with logit link and binomial distribution. Influenza season was treated as a random effect in the model. The odds of influenza vaccination among children with laboratory-confirmed influenza positive (cases) were compared to the odds of influenza vaccination among children who were tested influenza negative (controls). The VE was calculated as (1—adjusted odds ratio) × 100%. All potential confounding factors, such as age, gender, specimen collection date, or geographical region were initially evaluated. Only those factors that changed the crude odds ratio by ≥ 5% were included in the GLMM models to adjust VE. The point estimate of VE was considered significant when lower limit of the associated 95% confidence interval (CI) did not contain zero or negative value. In addition to overall VE estimated against any influenza viruses in entire children population, we estimated VE by influenza virus (sub)type in separate models [i.e., influenza A(H1N1)pdm09, influenza A(H3N2), or influenza B], and in stratified models by age category.

The characteristics of patients by the status of influenza testing result were compared by odds ratio and Chi-square test. The simple linear regression analysis was conducted to explore the potential relationship of VE by age [the average of each age subgroup (i.e., 6–23 months, 2–4 years, 5–8 years, 9–12 years, and 13–17 years]. A p < 0.05 was considered statistically significant.

All analyses were conducted using SAS Enterprise Guide 7.1(SAS Institute Inc., Cary, NC).

### Ethics

The present study was determined to be “Public Health Practice” and “Not Human Use Research”, and was exempted from review by the DoD Air Force Research Laboratory’s Institutional Review Board. Regulations or rules were followed to protect patient’s identity and health information.

## Results

### Patient characteristics

The characteristics of the patients overall and for each season are shown in Table 1. During the four influenza seasons from 2016–2017 to 2019–2020, a total of 9,385 children aged 6 months-17 years were identified for the VE analysis; among whom there were 3,768 (40.1%) children aged 6 months-4 years and 5,617 (59.9%) children aged 5–17 years. There were 1,804 (44.4%) vaccinated against influenza and 2,259 (55.6%) unvaccinated among 4,063 influenza-positive cases, while 3,146 (59.1%) vaccinated against influenza and 2,176 (40.9%) unvaccinated were among 5,322 influenza-negative controls (Table 1). Of the influenza-positive cases, influenza A(H1N1)pdm09, influenza A(H3N2), and influenza B were 928 (22.8%), 1,427 (35.1%), and 1,331 (32.8%), respectively, with the remaining being 365 (9.0%) non-subtyped influenza A and 12 (0.3%) influenza co-infection (Table 1).

**Table 1:** Characteristics of study population used for vaccine effectiveness analysis over four influenza seasons (2016–2017 to 2019–2020).

	Overall			2016–2017			2017–2018			2018–2019			2019–2020		
Characteristic	Cases (%)	Controls (%)	p-value	Cases (%)	Controls (%)	p-value	Cases (%)	Controls (%)	p-value	Cases (%)	Controls (%)	p-value	Cases (%)	Controls (%)	p-value
Gender															
Male	2124 (52.28)	2801 (52.63)	0.734	358 (52.03)	419 (52.18)	0.956	435 (50)	619 (53.04)	0.174	669 (54.48)	888 (52.30)	0.243	662 (51.84)	875 (52.90)	0.568
Female	1939 (47.72)	2521 (47.37)		330 (47.97)	384 (47.82)		435 (50)	548 (46.96)		559 (45.52)	810 (47.70)		615 (48.16)	779 (47.10)	
Age															
6–11 months	117 (2.88)	503 (9.45)	<0.001	19 (2.76)	74 (9.22)	<0.001	33 (3.79)	136 (11.65)	<0.001	21 (1.71)	133 (7.83)	<0.001	44 (3.45)	160 (9.67)	<0.001
12–23 months	194 (4.77)	843 (15.84)		24 (3.49)	108 (13.44)		51 (5.86)	182 (15.59)		54 (4.40)	275 (16.19)		65 (5.09)	278 (16.80)	
2–4 years	699 (17.20)	1412 (26.53)		89 (12.94)	219 (27.27)		162 (18.62)	314 (26.91)		241 (19.63)	438 (25.80)		207 (16.21)	441 (26.66)	
5–8 years	1416 (34.85)	1102 (20.71)		217 (31.54)	173 (21.54)		277 (31.84)	218 (18.68)		442 (35.99)	374 (22.03)		480 (37.59)	337 (20.37)	
9–12 years	947 (23.31)	727 (13.66)		181 (26.31)	108 (13.45)		183 (21.03)	160 (13.71)		287 (23.37)	256 (15.08)		296 (23.18)	203 (12.27)	
13–17 years	690 (16.98)	735 (13.81)		158 (22.97)	121 (15.07)		164 (18.85)	157 (13.45)		183 (14.90)	222 (13.07)		185 (14.49)	235 (14.21)	
Month of illness															
November	89 (2.19)	299 (5.62)	<0.001	1 (0.15)	31 (3.86)	<0.001	9 (1.03)	62 (5.31)	<0.001	0 (0)	0 (0)		79 (6.19)	206 (12.45)	<0.001
December	360 (8.86)	753 (14.15)		40 (5.81)	103 (12.83)		71 (8.16)	148 (12.68)		26 (2.12)	163 (9.60)		223 (17.46)	339 (20.50)	
January	1093 (26.90)	1290 (24.24)		132 (19.19)	170 (21.17)		347 (39.89)	334 (28.62)		208 (16.94)	423 (24.91)		406 (31.79)	363 (21.95)	
February	1566 (38.54)	1473 (27.68)		239 (34.74)	208 (25.90)		300 (34.48)	347 (29.73)		584 (47.56)	504 (29.68)		443 (34.69)	414 (25.03)	
March	782 (19.25)	1156 (21.72)		212 (30.81)	222 (27.65)		109 (12.53)	198 (16.97)		335 (27.28)	404 (23.79)		126 (9.87)	332 (20.07)	
April	173 (4.26)	351 (6.60)		64 (9.30)	69 (8.59)		34 (3.91)	78 (6.68)		75 (6.11)	204 (12.01)		0 (0)	0 (0)	
Geographic region															
Eastern US	2107 (51.86)	2684 (50.43)	0.171	459 (66.72)	487 (60.65)	0.015	501 (57.59)	675 (57.84)	0.908	493 (40.15)	737 (43.40)	0.078	654 (51.21)	785 (47.46)	0.044
Western US	1956 (48.14)	2638 (49.57)		229 (33.28)	316 (39.35)		369 (42.41)	492 (42.16)		735 (59.85)	961 (56.60)		623 (48.79)	869 (52.54)	
Vaccine status															
Vaccinated	1804 (44.40)	3146 (59.11)	<0.001	211 (30.67)	379 (47.20)	<0.001	320 (36.78)	642 (55.01)	<0.001	716 (58.31)	1113 (65.55)	<0.001	557 (43.62)	1012 (61.19)	<0.001
Unvaccinated	2259 (55.60)	2176 (40.89)		477 (69.33)	424 (52.80)		550 (63.22)	525 (44.99)		512 (41.69)	585 (34.45)		720 (56.38)	642 (38.81)	
Influenza															
A(H1N1)pdm09	928 (22.84)	0 (0)	/	9 (1.31)	0 (0)	/	149 (17.13)	0 (0)	/	273 (22.23)	0 (0)	/	497 (38.92)	0 (0)	/
A(H3N2)	1427 (35.12)	0 (0)		477 (69.33)	0 (0)		370 (42.53)	0 (0)		567 (46.17)	0 (0)		13 (1.02)	0 (0)	
A/not Subtyped	365 (8.98)	0 (0)		0 (0)	0 (0)		2 (0.23)	0 (0)		355 (28.91)	0 (0)		8 (0.63)	0 (0)	
B	1331 (32.76)	0 (0)		198 (28.78)	0 (0)		341 (39.20)	0 (0)		33 (2.69)	0 (0)		759 (59.44)	0 (0)	
Dual Influenza	12 (0.30)	0 (0)		4 (0.58)	0 (0)		8 (0.92)	0 (0)		0 (0)	0 (0)		0 (0)	0 (0)	
Non-influenza	0 (0)	5322 (100)		0 (0)	803 (100)		0 (0)	1167 (100)		0 (0)	1698 (100)		0 (0)	1654 (100)	

### Confounding factors assessment

Among all potential confounders examined, month of specimen collected and age were the only variables that changed the crude odds ratio by ≥ 5%. Therefore, month of specimen collected (i.e., one-month period from November to April of the following year) and the age (i.e., 6–23 months, 2–4 years, 5–8 years, 9–12 years, and 13–17 years) were included in the models to estimate overall VE and the VE stratified by influenza type/subtypes or age (sub)groups. Additionally, the age strata (i.e., 6–11 months and 1–2 years) was included in the model to estimate the VE for children aged 6–23 months.

### VE overall and by age groups

Adjusted VE against laboratory-confirmed influenza for medically attended children aged 6 months-17 years, was 42% (95% CI: 37–47%) overall, including 55% (95% CI: 47–61%) against influenza A(H1N1)pdm09, 37% (95% CI: 28–45%) against influenza A(H3N2), and 49% (95% CI: 41–55%) against influenza B (Table 2). By age groups, the adjusted VE for children aged 6 months-4 years was 48% (95% CI: 39–55%) against any influenza viruses, including 61% (95% CI: 50–70%) against influenza A(H1N1)pdm09, 39% (95% CI: 23–52%) against influenza A(H3N2), and 58% (CI: 45–69%) against influenza B. For children aged 5–17 years, the adjusted VE was 39% (95% CI: 32–46%) against any influenza viruses, including 51% (95% CI: 40–60%) against influenza A(H1N1)pdm09, 37% (95% CI: 26–46%) against influenza A(H3N2), and 45% (35–53%) against influenza B (Table 2).

**Table 2:** Adjusted vaccine effectiveness overall and by age group in children aged 6 months-17 years.

	Unvaccinated		Vaccinated			
	Total	Cases (%)	Total	Cases (%)	VE (%)^a^	95% CI (%)
6 months-4 years						
Influenza A(H1N1)pdm09	1108	153 (13.81)	1958	155 (7.92)	61	50–70
Influenza A(H3N2)	1133	178 (15.71)	1987	184 (9.26)	39	23–52
Influenza B	1088	133 (12.22)	1921	118 (6.14)	58	45–69
Any influenza^b^	1439	484 (33.63)	2329	526 (22.58)	48	39–55
5–17 years						
Influenza A(H1N1)pdm09	1588	367 (23.11)	1596	253 (15.85)	51	40–60
Influenza A(H3N2)	1840	619 (33.64)	1789	446 (24.93)	37	26–46
Influenza B	1910	689 (36.07)	1734	391 (22.55)	45	35–53
Any influenza^b^	2996	1775 (59.25)	2621	1278 (48.76)	39	32–46
6 months-17 years						
Influenza A(H1N1)pdm09	2696	520 (19.29)	3554	408 (11.48)	55	47–61
Influenza A(H3N2)	2973	797 (26.81)	3776	630 (16.68)	37	28–45
Influenza B	2998	822 (27.42)	3655	509 (13.93)	49	41–55
Any influenza^b^	4435	2259 (50.94)	4950	1804 (36.44)	42	37–47

^a^ VE: vaccine effectiveness, adjusted for months of specimen collected (November, December, January, February, March, and April), and age (6–23 months, 2–4 years, 5–8 years. 9–12 years, 13–17 years).

^b^ Including influenza A(H1N1)pdm09, influenza A(H3N2), influenza B, influenza A/not subtyped, and influenza co-infection.

### VE by age subgroups

**Fig 1:**
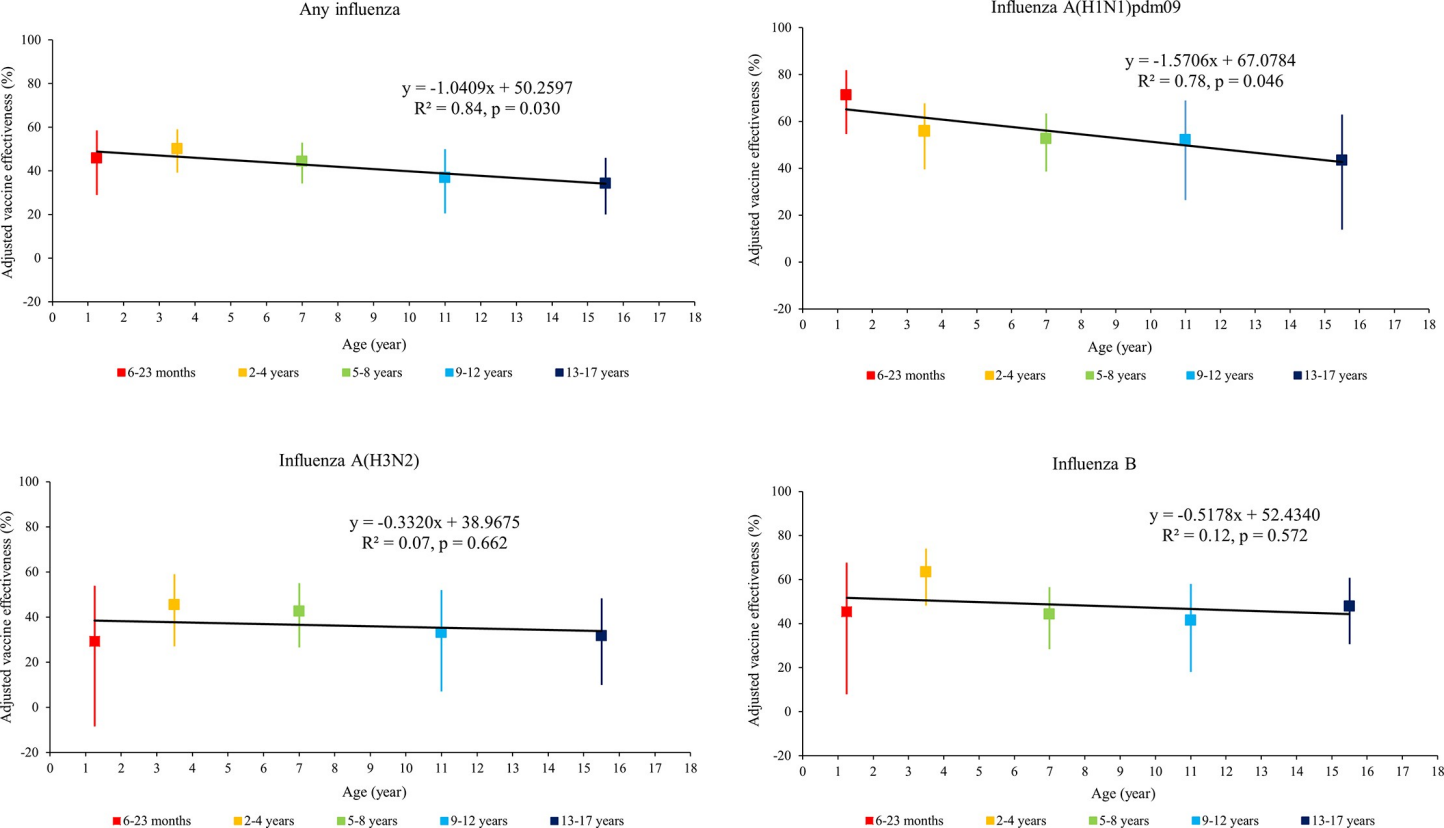
a. Inactivated influenza vaccine effectiveness against any influenza. b. Inactivated influenza vaccine effectiveness against influenza A(H1N1)pdm09. c. Inactivated influenza vaccine effectiveness against influenza A(H3N2). d. Inactivated influenza vaccine effectiveness against influenza B.

The adjusted VE against laboratory-confirmed influenza (sub)types for children by age subgroups are shown in Fig 1A–1D. The adjusted overall VE were 46% (95% CI: 29–59%) for children aged 6–23 months, and 50% (95% CI: 39–59%) for children aged 2–4 years. Thereafter, the adjusted overall VE declined linearly with increasing age, from 44% (95% CI: 34–53%) in children aged 5–8 years, to 37% (95% CI: 21–50%) in children aged 9–12 years, and then to 34% (95% CI: 20–46%) in children aged 13–17 years (Table 3; Fig 1A, R^2^ = 0.84, p = 0.030). By influenza (sub)type, it was found that the adjusted VE (71%; 95% CI: 55–82%) was the highest against influenza A(H1N1)pdm09 at 6–23 months of age, and decreased linearly with increasing age thereafter (Table 3; Fig 1B, R^2^ = 0.78, p = 0.046). In contrast, with increasing age, the adjusted VE against influenza A(H3N2) did not linearly declined (Table 3; Fig 1C, R^2^ = 0.07, p = 0.662). Similarly, no linear relationship was found between the adjusted VE against influenza B and children age (Table 3; Fig 1D, R^2^ = 0.12, p = 0.572).

**Table 3:** Adjusted vaccine effectiveness by age subgroup in children aged 6 months-17 years.

	Unvaccinated		Vaccinated			
	Total	Case (%)	Total	Case (%)	VE (%)^a^	95% CI (%)
6–23 months						
Influenza A(H1N1)pdm09	476	50 (10.50)	972	52 (5.35)	71	55–82
Influenza A(H3N2)	472	46 (9.75)	980	60 (6.12)	29	-8-54
Influenza B	455	29 (6.37)	969	49 (5.06)	45	8–68
Any influenza^b^	556	130 (23.38)	1101	181 (16.44)	46	29–59
2–4 years						
Influenza A(H1N1)pdm09	632	103 (16.3)	986	103 (10.45)	56	40–68
Influenza A(H3N2)	661	132 (19.97)	1007	124 (12.31)	45	27–59
Influenza B	633	104 (16.43)	952	69 (7.25)	63	48–74
Any influenza^b^	883	354 (40.09)	1228	345 (28.09)	50	39–59
5–8 years						
Influenza A(H1N1)pdm09	684	213 (31.14)	794	163 (20.53)	53	39–63
Influenza A(H3N2)	718	247 (34.4)	837	206 (24.61)	43	27–55
Influenza B	749	278 (37.12)	813	182 (22.39)	44	28–57
Any influenza^b^	1251	780 (62.35)	1267	636 (50.20)	44	34–53
9–12 years						
Influenza A(H1N1)pdm09	364	83 (22.8)	334	44 (13.17)	52	26–69
Influenza A(H3N2)	424	143 (33.73)	397	107 (26.95)	33	7–52
Influenza B	450	169 (37.56)	382	92 (24.08)	41	18–58
Any influenza^b^	708	427 (60.31)	575	285 (49.57)	37	21–50
13–17 years						
Influenza A(H1N1)pdm09	540	71 (13.15)	468	46 (9.83)	43	14–63
Influenza A(H3N2)	698	229 (32.81)	555	133 (23.96)	32	10–48
Influenza B	711	242 (34.04)	539	117 (21.71)	48	31–61
Any influenza^b^	1037	568 (54.77)	779	357 (45.83)	34	20–46

^a^ VE: vaccine effectiveness, adjusted for months of specimen collecting date (November, December, January, February, March, and April), for children aged 6–23 months, adjusted for age (6–11 months, 12–23 months) additionally.

^b^ Including influenza A(H1N1)pdm09, influenza A(H3N2), influenza B, influenza A/not subtyped, and influenza co-infection.

### VE of special pediatric population (ages of 6–11 months and 12–23 months)

Further age-stratified VE was estimated in children aged 6–23 months (Table 4). For children aged 6–11 months, the adjusted VE was 33% (95% CI: -2-56%) against any influenza viruses, including 52% (95% CI: 6–76%) against influenza A(H1N1)pdm09, 32% (95% CI: -34-66%) against influenza A(H3N2), and 5% (CI: -121-59%) against influenza B. By contrast, the adjusted VE was 54% (95% CI: 34–67%) against any influenza viruses, including 81% (95% CI: 65–90%) against influenza A(H1N1)pdm09, 31% (95% CI: -21-61%) against influenza A(H3N2), and 64% (95% CI: 29–82%) against influenza B in children aged 12–23 months (Table 4).

**Table 4:** Adjusted vaccine effectiveness in children aged 6–11 months and 12–23 months.

	Unvaccinated		Vaccinated			
	Total	Case (%)	Total	Case (%)	VE (%)^a^	95% CI (%)
6–11 months						
Influenza A(H1N1)pdm09	233	22 (9.44)	312	20 (6.41)	52	6–76
Influenza A(H3N2)	234	23 (9.83)	310	18 (5.81)	32	-34-66
Influenza B	221	10 (4.52)	310	18 (5.81)	5	-121-59
Any influenza^b^	269	58 (21.56)	351	59 (16.81)	33	-2-56
12–23 months						
Influenza A(H1N1)pdm09	243	28 (11.52)	660	32 (4.85)	81	65–90
Influenza A(H3N2)	238	23 (9.66)	670	42 (6.27)	31	-21-61
Influenza B	234	19 (8.12)	659	31 (4.70)	64	29–82
Any influenza^b^	287	72 (25.09)	750	122 (16.27)	54	34–67

^a^ VE: vaccine effectiveness, adjusted for months of specimen collecting date (November, December, January, February, March, and April).

^b^ Including influenza A(H1N1)pdm09, influenza A(H3N2), influenza B, influenza A/not subtyped, and influenza co-infection.

## Discussion

We report the pooled estimates of VE against medically attended, laboratory-confirmed influenza among children aged 6 months-17 years over four influenza seasons (2016–2017 through 2019–2020). Significant protection of IIV was found against any influenza (42%), while by influenza (sub)type, the protection was higher for influenza A(H1N1)pdm09 and influenza B, and lower for influenza A(H3N2) (Table 2). Moreover, higher overall VE against any influenza viruses was found in children aged 6 month-4 years vs. 5–17 years. The age-stratified analysis indicated the overall VE estimates against any influenza and the VE estimates against A(H1N1)pdm09 declined linearly as age increased (Table 3 and Fig 1A and 1B).

Recently, a number of meta-analysis have been conducted to systematically review and summarize the effectiveness of influenza vaccine of published studies in a single influenza season or over multiple influenza seasons [10–12]. Belongia et al. [10] found, in their analysis of the pediatric population (<20 years old) from 2004 to 2015, that a pooled VE against medically attended influenza in children was 69% (95% CI: 49–81%) for influenza A(H1N1)pdm09, 43% (95% CI: 28–55%) for influenza A(H3N2), and 56% (95% CI: 38–69%) for influenza B. Chung et al. [11] combined data from 5 studies in the US from 2013–2014 through 2015–2016 influenza seasons among outpatients aged 2 to 17 years, and their analysis showed that the VE against any types of medically attended, laboratory-confirmed influenza was 51% (95% CI: 47–54%). In another meta-analysis for children aged 6 months-17 years that included data from outside the US (i.e., Canada, Finland, Germany, and the United Kingdom), in the 2016–2017 influenza season, the consolidated VE estimates against influenza were 47% (95% CI: 29–61) overall, 46% (95% CI: 33–56%) against influenza A(H3N2), and 58% (95% CI; 17–79%) against influenza B [12]. By comparison, the influenza VE estimates in children aged 6 months-17 years in the present study, either overall or by (sub)types, appeared to be lower but comparable (Table 2).

Kalligeros et al. [13] showed that influenza VE against influenza-related hospitalization in children was 57% (95% CI: 49–65%) overall, including 74% (95% CI: 55–93%) against influenza A(H1N1)pdm09, 41% (95% CI: 26–56%) against influenza A(H3N2), and 51% (95% CI: 42–60%) against influenza B. By contrast, we found in outpatient settings influenza vaccine offered lower protection in children overall or against influenza A(H1N1)pdm09, but comparable protection against influenza A(H3N2) or influenza B. Although VE estimates obtained from outpatient and inpatient settings might be expected to differ, Feng et al. [14] found that there were no differences in the VE estimates between outpatient and inpatient settings based on the published studies using the test-negative design.

By age groups (6 months-4 years and 5–17 years), previous studies have shown influenza vaccines are effective in children aged 6 months-4 years against laboratory-confirmed influenza [15–17]. The US studies usually separated children into two age categories (6 months-8 years and 9–17 years) to measure the effectiveness of influenza vaccination to prevent outpatient visits for laboratory-confirmed influenza infections [18–23], with an exception that during 2017–2018 influenza season, children were separated into two age categories of 6 months-4 years and 5–17 years [24]. During 3 consecutive influenza seasons over 2016–2017 through 2018–2019, children aged 6 months-4 years (or 6 months-8 years) had higher VE estimates against any influenza, compared to those in children aged 5–17 years (or 9–17 years) in each individual study [22–24]. In the present study, the pooled VE against any influenza was 48% (95% CI: 39–55%) in children aged 6 months-4 years and 39% (95% CI: 32–46%) in children aged 5–17 years. Similarly, higher VE estimates were observed in children aged 6 months-4 years vs. 5–17 years. It appeared that when vaccinated, children at younger age have stronger protection against influenza, although inconsistent observations have been reported [19, 21].

Children aged <2 years are at high risk of developing serious influenza-related complications [25]. Generally, it is of public health interest to evaluate the effectiveness of influenza vaccination in this special pediatric population comprising infants and young children <2 years old of age. In the present study, there were >4000 identified cases of influenza over four consecutive influenza seasons. Our large sample size provided an opportunity to further compare age-specific VE by influenza (sub)type. It was shown that the adjusted overall VE (46%) in children aged 6–23 months was low but comparable, in comparison to the VE (ranging from 51 to 86%) against any laboratory-confirmed influenza reported previously in published studies [15, 16, 26]. Our findings demonstrated inactivated influenza vaccine was moderately effective against any influenza in children aged 6–23 months [27].

In the separate VE analysis for children aged 6–23 months, we found there were great differences of the VE estimates in children aged between 6–11 months and 1–2 years, with the exception of similar low and not-significant VE against influenza A(H3N2). For children aged 6–11 months, both overall VE and the VE against influenza A(H3N2) and influenza B were low and non-significant; moreover, 95% CI (6–76%) of the VE against influenza A(H1N1)pdm09 was wide, with lower limit for the mean being low close to zero. By comparison, higher overall VE and the VE against influenza A(H1N1)pdm09 and influenza B were observed in age 12–23 months. Consistently, several studies in Japan [26, 28–30], which performed VE analysis each season individually throughout the four seasons from 2013–2014 to 2016–2017, or via a combination of three-season data from 2013–2014 to 2015–2016, have shown low and non-significant effectiveness of influenza vaccination for children aged 6–11 months. Influenza vaccination has been recommended for infants starting 6 months old in the US [5]. Nevertheless, our findings support the view that influenza vaccination should not be strongly recommended for children aged 6–11 months [29, 30]. Further research efforts should be made to elicit immune response in infants 6–11 months of age to influenza vaccination, thus providing more robust evidence to substantiate those findings.

The overall VE against any influenza declined linearly with increasing age (Fig 1A). The pattern of decreasing VE as age increases may suggest a role of immune history in shaping the vaccine response. There are evidences suggesting that repeated vaccination impacts vaccine protection against influenza virus, which might consequently alter VE estimates [31, 32]. The underlying immunologic mechanisms for the potential vaccine interference are not well understood [33]. Shinjoh et al. [30] attributed possibly a low VE in children aged 13–15 years to their strong immunity, due to the past history of natural influenza infection and influenza vaccination. Indeed, children aged <2 years often receive influenza vaccination for the first time in their lives, while as they grow older, there are more chances for them to experience influenza infection or receive repeated influenza vaccination.

The DoDGRS program collects, compiles and analyzes outpatient influenza information from DoD beneficiary year-round to monitor global influenza activity. Influenza VE have been evaluated annually via the test-negative case-control design [6–9]. In those individual analysis, sample size might be too small to permit accurate VE estimates for specific age category of interest. All DoDGRS data were collected using same or similar methods during the 2016–2017 through 2019–2020 influenza seasons, which allowed pooling for estimation of VE. Moreover, as shown above, pooling data increased the available sample size, thus allowed for an age-stratified evaluation of VE by influenza (sub)type. Nevertheless, while pooling data from multiple influenza seasons increases statistical power for analysis, it may mask important variation at the individual season level [33]. Indeed, there are season to season variations in circulating viruses, vaccine match, and host-virus immunological interactions [32]. In the present study, we conducted the analysis to estimate pooled VE using generalized mixed models, with influenza season being considered a random effect to account for the heterogeneity across the influenza seasons. Using a random effect approach does statistically adjust for the heterogeneity; however, there may still be residual heterogeneity across seasons [34]. Although there are statistical methodological challenges arising from pooling data, pooling individual data over multiple seasons permits effectiveness analysis of IIV among children, specifically for targeted age category, thus exploring the potential relationship between the estimated VE with those virus and host factors influencing VE against influenza viruses.

The VE estimates in the present study have been adjusted, using month of specimen collected and age, identified from potential confounders such as covariates being used, gender, and geographical region, etc. as covariate in generalized linear mixed models. However, due to lack of randomization that is inherent in any observational study, it is difficult to rule out unmeasured confounding factors as a possible alternative explanation for the findings. For instance, we were unable to obtain all information on past history of vaccine status and influenza infection in previous seasons. Thus, the models used in the present study did not account for prior exposure to influenza by repeated influenza vaccination or natural influenza infection in previous seasons. Therefore, it would be valuable to collect such patients’ data in future endeavors. In addition, our efforts to estimate the effect of vaccination rely on the DoD surveillance platform for data acquisition. There are limitations worth mentioning with regard to the DoD surveillance data collection. When ascertaining vaccine status based on the self-reported questionnaire, potential non-differential misclassification could occur. Moreover, the specimens collected from the sentinel and participating sites in DoDGRS were through routine outpatient clinical care. Before evaluating the validity of using such routinely collected data in VE analysis, possibility of potential selection bias could not be completely ruled out.

In conclusion, the present study demonstrated moderate effectiveness of inactivated influenza vaccination in children against medically attended, laboratory-confirmed influenza infections. We found the VE was higher against influenza A(H1N1)pdm09 and influenza B, but comparable against influenza A(H3N2) in children aged 6 months-4 years, when compared to those in children aged 5–17 years. Moreover, we observed low and non-significant VE against any influenza in children aged 6–11 months, which was consistent with the finding from the previous observational studies in Japan [26, 28–30]. Further research that includes assessment of host immune response to influenza vaccine in children 6–11 months of age is warranted, which would undoubtedly provide valuable information helping guide future vaccine policy recommendations.

## Supporting information

S1 Data (XLSX)
